# Progress and challenges in applying CRISPR/Cas techniques to the genome editing of trees

**DOI:** 10.48130/FR-2022-0006

**Published:** 2022-05-11

**Authors:** Solme Pak, Chenghao Li

**Affiliations:** State Key Laboratory of Tree Genetics and Breeding, Northeast Forestry University, Harbin 150040, China

**Keywords:** Clustered Regularly Interspaced Short Palindromic Repeats/CRISPR-associated protein, Forest tree, Gene editing, Genetic transformation, Ribonucleo-protein complex

## Abstract

With the advent of the Clustered Regularly Interspaced Short Palindromic Repeats (CRISPR)/CRISPR-associated protein (Cas) system, plant genome editing has entered a new era of robust and precise editing for any genes of interest. The development of various CRISPR/Cas toolkits has enabled new genome editing outcomes that not only target indel mutations but also enable base editing and prime editing. The application of the CRISPR/Cas toolkits has rapidly advanced breeding and crop improvement of economically important species. CRISPR/Cas toolkits have also been applied to a wide variety of tree species, including apple, bamboo, Cannabaceae, cassava, citrus, cacao tree, coffee tree, grapevine, kiwifruit, pear, pomegranate, poplar, ratanjoyt, and rubber tree. The application of editing to these species has resulted in significant discoveries related to critical genes associated with growth, secondary metabolism, and stress and disease resistance. However, most studies on tree species have involved only preliminary optimization of editing techniques, and a more in-depth study of editing techniques for CRISPR/Cas-based editing of tree species has the potential to rapidly accelerate tree breeding and trait improvements. Moreover, tree genome editing still relies mostly on Cas9-based indel mutation and *Agrobacterium*-mediated stable transformation. Transient transformation for transgene-free genome editing is preferred, but it typically has very low efficiency in tree species, substantially limiting its potential utility. In this work, we summarize the current status of tree genome editing practices using the CRISPR/Cas system and discuss limitations that impede the efficient application of CRISPR/Cas toolkits for tree genome editing, as well as future prospects.

## Introduction

Trees are essential components of most ecosystems that play significant roles in lowering the atmospheric level of CO_2_, protecting biodiversity, and providing food and materials for human consumption. Ever-increasing demands for forest products, as well as concerns about global warming due to elevated CO_2_ levels, have increased the need for more efficient improvement of tree varieties. In the past, researchers and breeders have employed traditional approaches, including hybrid breeding, mutagenesis, and polyploid breeding, to achieve a variety of trait improvements and gain a better understanding of gene function. Traditional breeding approaches require tremendous time, and mutation screening is dramatically hindered by tree species' long generation time and complex genome polyploidy and heterozygosity. The advent of Clustered Regularly Interspaced Short Palindromic Repeats (CRISPR)/CRISPR-associated protein (Cas) genome editing technology has significantly accelerated plant breeding and functional genomics with high speed and precision.

The CRISPR/Cas approach involves an adaptive phage immunity system from archaea and bacteria. This system relies on a single RNA called "single guide RNA" (sgRNA) to guide DNA–RNA recognition and binding for sequence-specific nucleic acid cleavage and can be readily programmed to introduce DNA double-strand breaks (DSBs) at any desired target site at minimal cost^[1]^. For more than two decades, CRISPR/Cas systems were of interest mainly to microbiologists who investigated the unique mechanisms underlying the CRISPR/Cas adaptive immunity systems of prokaryotes. The potential for CRISPR/Cas systems to serve as genome editing tools was initially recognized in 2012; thereafter, they began to be applied to mammals and were developed into crucial tools for research and clinical applications such as gene therapy^[2,3]^. CRISPR/Cas systems have also been widely used in plants. CRISPR/Cas was first applied to plants in 2013^[4]^ and has subsequently been used in 45 plant genera from 24 different families, demonstrating the high efficiency, simplicity, and versatility of this system^[5]^. Various Cas9 variant proteins such as Cas12a (Cpf1), Cas13, and Cas14, as well as nuclease-deactivated Cas proteins (dCas9 or dCas12) fused with a base editor, prime editor, or other epigenomic modifier proteins, have been developed to enhance the versatility of CRISPR toolkits in mammals and plants^[3,6−8]^. Moreover, the requirement for a protospacer adjacent motif (PAM), which is a natural constraint on the flexibility of CRISPR toolkits, has recently been overcome. PAM-free nucleases such as SpRY have been generated through natural ortholog mining and protein engineering, enabling the targeting of virtually any site in genomic DNA^[9]^.

The application of CRISPR/Cas for gene function studies and trait improvement has been comparatively slow in tree species. In 2014, tree genome editing by the Cas9/sgRNA system was first reported in a citrus genome^[10]^, in which rapid and precise mutation of target genes was demonstrated within a short period (4 days) at a low efficiency (3.2%–3.9%). Significant effort has been made to improve the efficiency and stability of targeted mutagenesis in various tree species, such as apple^[11−13]^, bamboo^[14,15]^, Cannabaceae^[16]^, cassava^[17−19]^, citrus^[10,20−25]^, cacao tree^[26]^, coffee tree^[27]^, grapevine^[12,28−31]^, kiwifruit^[32,33]^, pear^[11]^, pomegranate^[34]^, poplar^[35−53]^, ratanjoyt^[54]^, and rubber tree^[55]^. These efforts have not only contributed to the establishment of CRISPR/Cas based-genome editing systems in trees^[10−14,17,23−25,27−30,32,35,37,43,48,53,55]^ but also promoted functional studies on tree trait genes that are crucial for tree breeding. Table 1 shows recent applications of CRISPR/Cas toolkits to tree genome editing. These practices all involve the sequential procedures of target gene selection, sgRNA design, and nuclease/sgRNA DNA vector construction or ribonucleoprotein preparation. These initial steps are followed by transformation, regeneration, screening of transformants, and mutation detection. Most practices have used the Cas9 nuclease, but there have been a few reported uses of the Cas12a nuclease^[23,53]^. *Agrobacterium*-mediated stable transformation has been the dominant transformation protocol, but despite its high efficiency, it is impractical owing to the current GMO (genetically modified organism) regulations in application. Transient transformation protocols such as the delivery of ribonucleo-protein (RNP) complexes can achieve transgene-free (non-GMO) genome editing and are therefore preferred. However, these approaches have much lower efficiency^[12,55]^, limiting their wide application. Overall, significant progress will be required to increase the utility of the CRISPR/Cas system in tree species. In this review, we summarize current progress in CRISPR/Cas-based tree genome editing and discuss limitations that affect the efficiency of this system, as well as future prospects.

**Table 1:** Application of the CRISPR/Cas system to tree genome editing.

Tree species	sgRNA design tool	Cas delivery enhancer	Promoter (sgRNA)	Promoter (Cas)	Multiplex targeting	Transformation protocol/explant	Regeneration protocol/time	Mutagenesis efficiency; mutation; mutants	Potential off-targets (Number; activity)	Reference
*Actinidia chinensis* cv. Hongyang	Cas-Designer	NLS	AtU6	35S	PTG	*Agrobacterium*/ leaf disc	CI-S; —	7.14%–91.67%; indel; biallelic, chimeric	4; N	[32]
*A. chinensis* cv. Hort16A	Geneious	NLS	AtU3, AtU6	Ubi, 35S	PTG	*Agrobacterium*/ leaf strip	CI-S-R; —	30%–75%; indel; biallelic, heterozygous	—	[33]
*Bambusa oldhamii*	—	NLS	OsU3	2× 35S	—	PEG (DNA)/protoplast	CI-S; 3 mon	12.5% (5/40); del&subs; —	—	[14]
*Citrus sinensis* cv. Valencia	—	NLS	35S	35S	—	Agroinfiltration/leaf	—	3.2%–3.9%; del; —	46; N	[10]
*C. sinensis* Osbeck	—	—	AtU6	35S	—	*Agrobacterium*/epicotyl	S-G; —	34.5% (38/110); del; biallelic, homozygous, heterozygous	11; 1-bp point mutations (5–10%)	[20]
*C. paradisi*	—	NLS	35S	35S	—	*Agrobacterium*/epicotyl	S-G; —	—; indel; —	85; N	[21]
				35S	—	*Agrobacterium*/leaf & epicotyl	S-G; —	23.8%–89.36%; indel; —	7; N	[22]
				Yao, 35S	—	*Agrobacterium*/epicotyl	S-G; —	42.8% (3/7); del; —	0	[23]
*Dendrocalamus latiflorus* Munro	—	—	OsU6	Ubi	—	PEG (DNA)/protoplast	CI-S-R; —	83.3%–100%; indel; homozygous, biallelic, heterozygous, chimeric	—	[15]
*Hevea brasiliensis*	—	—	—	—	—	PEG (RNP)/protoplast	—	3.74%–20.11%; indel; —	—	[55]
*Malus* × *domestic*a Bork.	CRISPOR	NLS	MdU3, MdU6	PcUbi4-2	Gateway cloning	*Agrobacterium*/young leaves	B; 6 mon	90% (27/30); indel&subs; biallelic chimeric	4; N	[11]
*Malus prunifolia* cv. Golden Delicious	CRISPR RGEN	NLS	—	—	—	PEG (RNP)/protoplast	—	6.9%; indel; —	—	[12]
*M. prunifolia* Borkh. 'Seishi'× M.	—	NLS	AtU6	2× 35S	—	*Agrobacterium*/leaf disc	S-R; 8 mon	10.9% (18/164); indel; homozygous, heterozygous, chimeric	0	[13]
*Coffea canephora* clone 197	—	NLS	AtU6	35S	Restriction enzyme ligation	*Agrobacterium*/ embryonic calli	SE; 18 mon	30.4% (28/92); indel; homozygous, heterozygous	0	[27]
*Manihot esculenta* cv. 60444; cv. TME 204	CRISPR-P	NLS	AtU6	35S	—	*Agrobacterium*/ embryonic calli	SE; —	19.1%–46.6%; indel&subs; homozygous, biallelic, heterozygous	—	[17]
*M. esculenta* cv. 60444	CRISPR-P	—	AtU6	2× 35S	Gibson assembly	*Agrobacterium*/ embryonic calli	SE; —	91%; indel; homozygous, biallelic, heterozygous, chimeric	5; single mutation for one off-target was detected	[18]
*Jatropha curcas*	CRISPR-P	NLS	AtU3	35S	—	*Agrobacterium*/cotyledon	CI-S; 4 mon	—; indel; homozygous	—	[54]
*Parasponia* *andersonii*	GPP sgRNA designer	NLS	AtU6	35S	—	*Agrobacterium*/ stem, petiole	CI-S-R; 4 mon	37.9%–88.9%; indel; biallelic, heterozygous	—	[16]
*Poncirus. trifoliata* L. Raf. × *C. sinensis* L. Osb	—	NLS	AtU6	Yao	—	*Agrobacterium*/epicotyl	S; 4 mon	85% (17/20); indel&subs; homozygous, monoallelic heterozygous	2; N	[24]
			35S, AtU6	35S	—	*Agrobacterium*/epicotyl	S-G; —	15.55%–79.67%; indel; homozygous	3; N	[25]
*Populus alba*	ZiFiT	NLS	AtU3, AtU6	35S	Golden Gate cloning	*Agrobacterium*/young leaves	CI-S-R; 3 mon	89%; del; —	—	[35]
84K poplar (*P. alba* × *P. glandulosa*)	CRISPR-P 2.0	NLS	AtU6	PcUbi4-2	—	*Agrobacterium*/leaf disc	—	—; indel; —	—	[36]
	—	NLS	AtU6	2× 35S	—	*Agrobacterium*/leaf disc	CI-S-R; —	6.7%–70%; del; biallelic, homozygous, heterozygous	—	[53]
*Populus tomentosa* Carr. clone 741	—	NLS	AtU3, AtU6	35S	Golden Gate cloning	*Agrobacterium*/leaf disc	CI-S-R; 3 mon	51.7% (30/59); indel&invers; biallelic homozygous, heterozygous	—	[37]
				—	Golden Gate cloning	*Agrobacterium*/leaf disc	CI-S-R; —	93.33%–100%; indel; —	—	[38]
				35S	Golden Gate cloning	*Agrobacterium*/leaf disc	CI-S-R; —	48% (12/25); indel; —	—	[39]
				35S	Golden Gate cloning	*Agrobacterium*/leaf disc	CI-S-R; —	—; indel; —	—	[40]
				35S	—	*Agrobacterium*/leaf disc	CI-S-R; —	—; indel; —	—	[41]
				35S	Golden Gate cloning	*Agrobacterium*/leaf disc	CI-S-R; —	—; indel; —	—	[42]
*Populus tremula* × *P. alba* clone 717	ZiFiT	NLS	AtU6	2× 35S	Restriction enzyme ligation	*Agrobacterium*/leaf, petiole, stem	CI-S-R; 18 wk	81.8% (479/585); indel&subs&invers; homozygous, biallelic, heterozygous, chimeric	5; N	[43]
	Geneious	NLS	MtU6	2× 35S	—	*Agrobacterium*/ leaf, stem	CI-S-R; —	100%; indel; biallelic	—	[44]
	Geneious	NLS	AtU6	PcUbi4-2	—	*Agrobacterium*/leaf disc	CI-S-R; 4–8 mon	—; indel; —	—	[45]
*Populus tremula* × *P. alba* INRA clone 717-1B4	—	—	MtU6	35S	—	*Agrobacterium*/ —	CI; —	—	—	[46]
	Geneious	NLS	MtU6	2× 35S	—	*Agrobacterium*/ hairy root	HR; —	40%; indel; —	5; N	[47]
	CRISPR-P 2.0	NLS	AtU6	Atact2	Golden Gate MoClo system assembly	*Agrobacterium*/leaf	HR; —	87.5% (14/16); indel; homozygous, biallelic, heterozygous	—	[48]
*Populus tremula* × *tremuloides* clone 353	ZiFiT	NLS	AtU6	2× 35S	Restriction enzyme ligation	*Agrobacterium*/leaf, petiole, stem	CI-S-R; 18 wk	88.8% (88/99); indel&subs&invers; homozygous, biallelic, heterozygous, chimeric	5; N	[43]
*Populus tremula* × *tremuloides* clone T89	CRISPR-P 2.0	NLS	—	35S	Golden Gate cloning	*Agrobacterium*/ —	—	—	—	[49]
*Populus trichocarpa* Nisqually-1	CRISPR-P 2.0	3x NLS	AtU6	2× 35S, PUbi4	Golden Gate cloning	*Agrobacterium*/leaf disc	CI-S; —	—; indel; —	—	[50]
	—	—	AtU6	2× 35S	—	*Agrobacterium*/stem	S-R; 17 wk	—; indel; —	—	[51]
	CRISPRdirect	—	AtU6	2× 35S	Golden Gate cloning	*Agrobacterium*/stem	S-R; 14 wk	75%–100%; indel; homozygous, biallelic, heterozygous, chimeric	8; N	[52]
*Punica granatum* L.	Cas-Designer	NLS	AtU6	AtUbi	Restriction enzyme ligation	*Agrobacterium*/ hairy root	HR; —	—; indel&subs; homozygous, biallelic, chimeric	—	[34]
*Pyrus communis* L. cv. Conference	CRISPOR	NLS	MdU3, MdU6	PcUbi4-2	Gateway cloning	*Agrobacterium*/young leaves	B; 7 mon	9% (5/54); indel&subs; biallelic chimerism	4; N	[11]
*Theobroma cacao*	Geneious	—	AtU6	35S	Golden Gate cloning	*Agrobacterium*/leaf, primary SE cotyledon	SE; —	27%; indel; —	9; 0.29–1.9% (off-target rate)	[26]
*Vitis vinifera* L. cv. Chardonnay	CRISPR-P	NLS	AtU6	35S	Golden Gate cloning	*Agrobacterium*/callus	S; —	100% (3/3); indel; heterozygous, chimeric	4; N	[28]
	CRISPR RGEN	NLS	—	—	—	PEG (RNP)/protoplast	—	0.1%; indel; —	—	[12]
*V. vinifera* cv. Thompson	CRISPR-P, CRISPR RGEN	NLS	AtU3, AtU6	2× 35S	Golden Gate cloning	*Agrobacterium*/ embryonic callus	SE; 12 mon	31% (22/72); large del; biallelic, monoallelic	6; N	[29]
*V. vinifera* L. cv. Neo Muscat	—	NLS	AtU6	PcUbi	—	*Agrobacterium*/ embryonic callus	CI-SE; 19–21 mon	2.7%–72.2%; indel; biallelic	3; N	[30]
*V. vinifera* cv. Chasselas × *V. berlandieri*	CRISPR-P	—	AtU6	35S	—	*Agrobacterium*/ embryonic cell	SE; —	66.6% (4/6); indel; biallelic, heterozygous, chimeric	2; N	[31]
Abbreviations: NLS, nuclear localization signal; AtU3/AtU6, Arabidopsis promoters for small nuclear RNA transcription; MtU3/MtU6, *Medicago truncatula* U3/6 promoters; MdU3/MdU6, *Malus domestica* U3/6 promoters; Cas, Cas nucleases; 35S, cauliflower mosaic virus (CaMV) 35S promoter; Ubi, ubiquitin promoter; PcUbi, *Petroselinum crispum* ubiquitin promoter; PTG, Polycistronic tRNA process system; GFP, green fluorescent protein; *Agrobacterium*, *Agrobacterium*-mediated T-DNA transfer; PEG (DNA), Polyethylene glycol–mediated DNA transfection; PEG (RNP), Polyethylene glycol–mediated ribonucleoprotein transfection; mon, month; wk, week; B, budding; CI, callus induction; S, shooting; R, rooting; G, grafting; SE, somatic embryogenesis; indel, insertion and deletion mutations; del, deletion mutation; subs, substitution mutation; invers, sequence inversion mutation; N, no activity detected; —, not mentioned.										

## The CRISPR/Cas system has rapidly advanced tree functional genomics and facilitated tree improvement

The CRISPR/Cas system is a major reverse genetics tool in functional genomics, and its application to trees has greatly promoted tree functional genomics. Various tree trait genes associated with early flowering^[33,46,49]^, growth^[15,31,36,45,54]^, symbiosis^[16]^, lignin biosynthesis associated with secondary growth for wood formation^[39,40,51,52]^, secondary metabolism^[34]^, and resistance to abiotic stresses^[36,40,50]^, diseases^[18−22,26,38,41,42]^, and herbivores^[47]^ have been functionally characterized by CRISPR/Cas techniques.

Since the application of CRISPR/Cas techniques to trees, tree breeding and trait improvement have been accelerated in parallel with rapid progress in tree functional genomics. In poplars, important woody species with high economic and ecological value, CRISPR/Cas9-mediated targeted mutagenesis has been used together with other genetic approaches to characterize the roles of important wood formation-related genes such as *PtoMYB156*^[39]^, *PtoMYB170*^[40]^, *Atypical aspartic protease* (*PtAP66*)^[51]^, and *cellulose synthase* (*PtrCesA*)^[52]^, highlighting their potential utility for enhancing the productivity and quality of wood. Functional characterization of *PdNF-YB21*^[36]^, *PdGNC*^[45]^, *LHY2*^[46]^, and *BRC1*^[49]^ by CRISPR/Cas9 and other genetic tools suggested that these genes have crucial roles in the regulation of root growth, photosynthesis, and seasonal growth cessation and therefore showed great potential for the breeding of fast-growing poplars. Transcription factors such as PtrABRE1^[50]^, PtoMYB170^[40]^, and PdNF-YB21^[36]^ have been demonstrated to regulate the response of poplar to drought stress, suggesting that they may be useful for breeding drought-resistant poplars. CRISPR/Cas9-mediated loss-of-function mutation has also contributed to the characterization of the MYB115 transcription factor^[38]^, the histone H3K9 demethylase gene *JMJ25*^[41]^, the PtrWRKY18 and PtrWRKY35 transcription factors^[42]^, and salicyl benzoate UDP-glycosyltransferase^[47]^, which are anti-fungal and anti-herbivore factors that are important for breeding fungal disease-resistant or herbivore-resistant poplars. In citrus, a widely cultivated fruit tree, the CRISPR/Cas system has been used to engineer canker disease-resistant varieties by targeting the promoter region of the disease susceptibility gene *CsLOB1*^[20−22]^. In cassava, a woody shrub extensively cultivated for its edible starchy tuberous root, two different approaches have been used to improve resistance to viral diseases. One involved knockout of the host *eIF4E* gene, which is crucial for interaction with viral genome-linked protein (VPg)^[18]^, and the other used CRISPRi (CRISPR interference) to target viral ACMV (African cassava mosaic virus) DNA A^[19]^. In cacao tree, the source of cocoa, CRISPR/Cas9 demonstrated its potential to enable the development of pathogen-resistant cacao varieties through editing of the *Non-Expressor of Pathogenesis-Related 3* (*TcNPR3*) gene^[26]^. In kiwifruit, a recently domesticated fruit tree species, the wild-type traits of perennial growth, non-compactness, long juvenility, and axillary flowering have hampered fruit development and productivity. CRISPR/Cas9-mediated mutation of the kiwifruit *CEN*-like gene *AcCEN4* or *AcCEN* transformed the wild-type plant into a compact plant with rapid terminal flowering, and the engineered kiwifruit plants were amenable to indoor farming and cultivation as annuals^[33]^. In grapevine, the source of grapes for direct consumption and fermentation into wine, CRISPR/Cas9-mediated knockout of the strigolactone (SL) biosynthesis genes *CCD7/8* enhanced shoot branching, showing the potential to increase grape productivity^[31]^.

## Workflow for tree genome editing using the CRISPR/Cas system

### Target gene selection and sgRNA design

The initial step of CRISPR/Cas genome editing is the design of sgRNAs against selected target genes, and well-designed sgRNAs are critical to editing success. Ideally, an sgRNA targeting sequence will have perfect homology to the target DNA, with no homology elsewhere in the genome. Online bioinformatic tools such as Cas-Designer^[32,34]^, CRISPOR^[11]^, CRISPR-P^[17,18,28,29,31,36,48−50,54]^, CRISPR RGEN^[12,29,52]^, and ZiFiT^[35,37−43]^, as well as the offline software tool Geneious^[33,44,45,47]^, have been used to design target-specific sgRNAs. However, the low availability of complete genome data for tree species has typically caused issues when attempting to assess on-target efficiency and potential off-targeting.

Recent efforts to sequence the genomes of trees have dramatically improved the design of sgRNAs. The TreeGenes database (https://treegenesdb.org) is a comprehensive resource for forest tree genomics that now includes complete genome sequences of 38 species and 3,920,817 transcriptome sequences from 263 tree species. Using these tree genomic resources and sgRNA design tools, it is now easy to design sgRNAs that are highly specific to any target DNA sequence and to predict on-target efficiency as well as off-target sites. Many of these tree genomic resources have already been uploaded to sgRNA design tools. Among the sgRNA design tools^[56]^, Cas-OFFinder, CCTop, CHOPCHOP, CRISPOR, CRISPRdirect, CRISPR-P v2.0, and E-CRISP now incorporate genome data from several tree species, enabling the genome-wide design of sgRNAs (see Supplemental Table S1). The genome data of 28 tree species are now available, including *Actinidia chinensis*, *Actinidia eriantha*, *Carica papaya*, *Citrus clementina*, *Citrus sinensis*, *Coffea canephora*, *Diospyros kaki*, *Eucalyptus grandis*, *Juglans macrocarpa*, *Juglans regia*, *Malus domestica*, *Manihot esculenta*, *Musa acuminata*, 717 hybrid poplar, *Populus alba* (sPta717 v2), *Populus alba* × *Populus tremula* var. glandulosa, *Populus deltoides*, *Populus tremula*, *Populus tremula* × *alba*, *Populus tremula* × *tremuloides*, *Populus tremuloides*, *Populus trichocarpa*, *Prunus avium*, *Prunus persica*, *Pyrus* × *bretschneideri*, *Ricinus communis*, *Theobroma cacao*, and *Vitis vinifera*. Cas-OFFinder, CCTop, CHOPCHOP, CRISPOR, and CRISPRdirect users can now also send requests for the addition of new genome data specific to their research, as long as the genome data are present in TreeGenes or other genome databases, including Ensembl, NCBI, and Phytozome. For tree species without sequenced genomes, the use of CRISPR still relies on gene cloning to obtain the target gene sequence (typically only exons), and off-target sites cannot be reliably predicted.

The sgRNA design tools also support the identification of sgRNAs without a genome sequence, although only cleavage efficiency is scored.

Several studies have shown that large genome size, high polyploidy and heterozygosity, and abundant single nucleotide polymorphisms (SNPs) in tree genomes cause significant problems in the design of highly target-specific sgRNAs. The frequent occurrence of SNPs in tree genomes (as many as 1 per 100 bp) can completely abolish cleavage of the target gene when they exist in the target sequence or the PAM sequence^[44]^. Recent efforts to sequence more tree genomes will undoubtedly remove these limitations and ensure more precise genome editing of tree species.

### Construction and transformation of the CRISPR/Cas reagent

Once sgRNAs are designed against target genes, they are inserted into plasmid vectors that contain the DNA sequence encoding a nuclease, such as Cas9, or directly mixed with the nuclease protein prior to transformation into tree explants. Target-specific nuclease tools such as Cas9/sgRNAs can be delivered via plasmid binary vectors or RNP complexes.

The most widely used form of CRISPR/Cas reagent is the *Agrobacterium* Ti plasmid binary vector, which harbors sequences of the Cas9 nuclease gene and designed sgRNAs in the T-DNA portion. During the construction of T-DNA vectors containing the Cas nuclease/sgRNAs expression cassette, the nuclear localization signal (NLS) has typically been fused to the Cas nuclease to enhance proper transport of Cas nuclease into the nucleus. Because the Cas9 nuclease is bacterial in origin, its codons are typically optimized for eukaryotic translation^[35−47,49,50]^. Promoters such as CaMV 35S, ubiquitin, and U3/6 small nuclear RNA (snRNA) have been used to drive the transcription of Cas nuclease genes and sgRNAs. The CaMV 35S promoter and ubiquitin promoter are strong constitutive promoters that drive *Cas9* gene expression broadly. This promoter is often used as a form of dual promoter to enhance *Cas9* gene transcription. Some studies have also used the meristem-specific Yao promoter to improve Cas nuclease expression efficiency^[23,24]^. For sgRNA transcription, U3/6 snRNA promoters have typically been employed. These promoters require A/G to be the first nucleotide at the transcription start site, which limits their utility^[10,21−23,25]^. Targeting of multiple genes or multiple sites in a gene has been performed using a multiple sgRNA cassette in a single CRISPR/Cas construct^[11,18,26−29,32−35,37−40,42,43,48−50,52]^, and the efficiency of multiplex gene editing has been improved by the use of a polycistronic tRNA-sgRNA cassette (PTG)^[32,33]^.

The use of a ribonucleoprotein complex with Cas nuclease protein and sgRNAs has been reported in some studies^[12,55]^. RNPs work transiently and then disappear, limiting potential off-targeting that can occur during prolonged Cas9 activity and enabling transgene-free genome editing that can avoid GMO regulation. The use of RNPs can also reduce the time required for Cas9 to be transcribed and translated in the Ti plasmid vector. Moreover, unlike plasmid vectors, RNP construction does not require codon optimization and species- and tissue-specific promoters.

The delivery of CRISPR/Cas reagents has mainly been achieved by *Agrobacterium*-mediated T-DNA integration into the genome. This stable transformation approach has been shown to result in highly efficient mutagenesis, but prolonged expression of the Cas9 nuclease has the potential to create off-target effects. In addition, delivery and further integration of the transgene is not favorable in the current regulations in application. Alternatively, transient expression approaches such as RNP transfection have been used to reduce off-targeting and achieve transgene-free genome editing, but they typically result in much lower editing efficiency^[12]^. The explants used for transformation include mostly the juvenile leaf, stem, petiole, embryogenic callus, and occasionally protoplasts. As the cell wall–free protoplast can easily take up exogenous transformation materials such as DNA or RNP, it is regarded as the "ideal" explant tissue for direct transformation approaches, including PEG (polyethylene glycol)-mediated transfer, electroporation, liposome-mediated transfer, biolistic bombardment, and others. However, it has only been reported in a few tree species^[12,14,15,55]^.

### Tissue regeneration, transformant selection, and mutation identification

In general, transformation is immediately followed by sequential tissue culture phases such as callus induction, shooting, and rooting. With the aid of tissue culture techniques, mutant cells can be readily cloned and then regenerated into whole plants. Stable inheritance of T-DNA containing CRISPR/Cas constructs within cells is critical for successful targeted mutagenesis, but could be subjected to the GMO regulations. RNP transfection has therefore been used as an alternative to avoid these issues and potentially reduce off-target effects^[12,55]^. Because all cells of the explant undergo regeneration under tissue culture conditions, selection markers such as antibiotic resistance genes (*NPTII* or *HPTII*) or reporter genes (*GUS* or *GFP*) have been used to identify transformants among regenerated plants. Mutations in the site of target genes and potential off-targets can then be detected by PCR and direct sequencing of the target gene amplicons. In most studies, no off-targeting has been detected even after stable transformation, despite the presence of potential off-targets (Table 1). Various kinds of on-target mutagenesis have been found, including biallelic, homozygous, heterozygous, and chimeric mutations.

## Current status of tree genome editing using the CRISPR/Cas system

**Figure 1:**
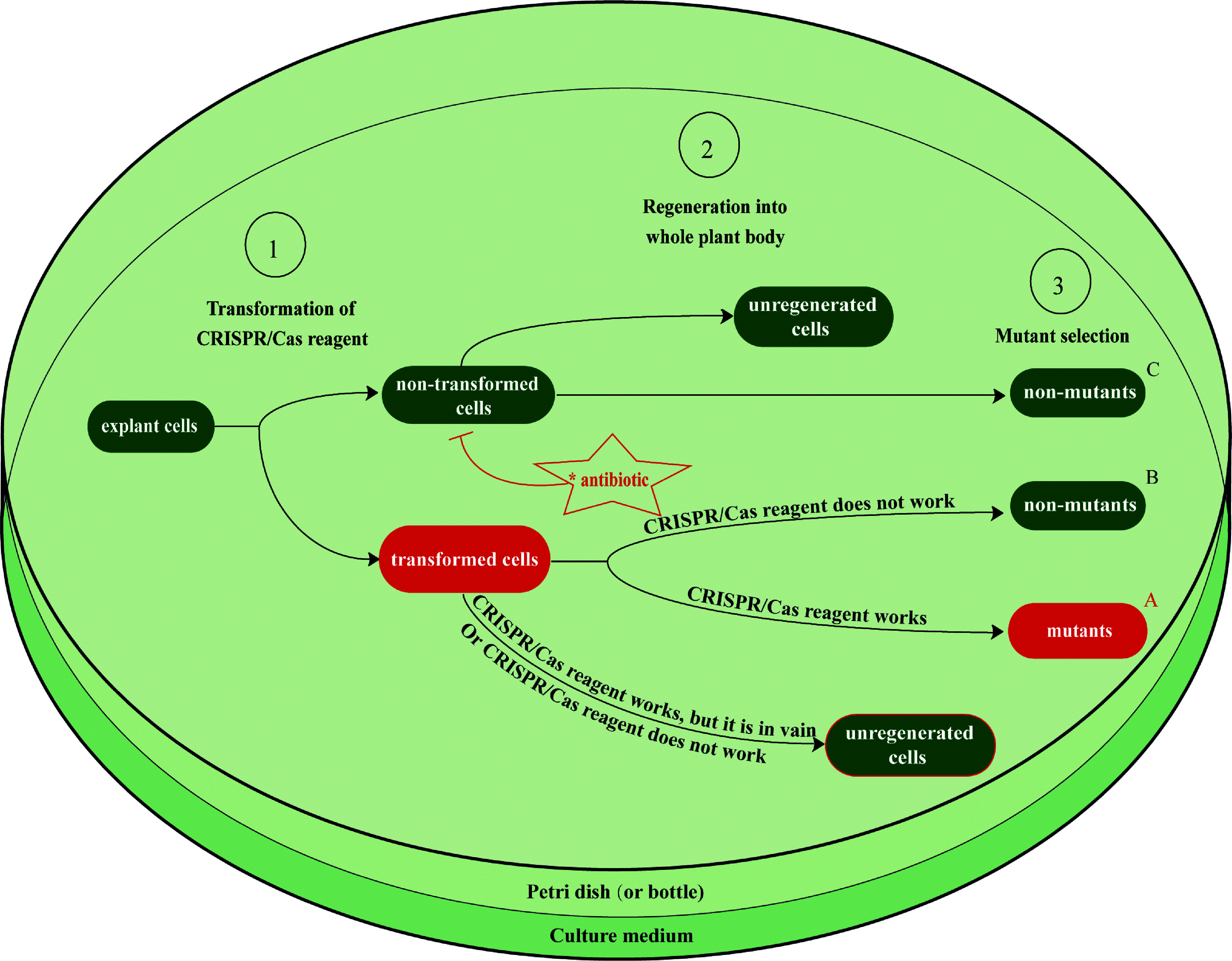
Schematic diagram for the wet-lab workflow of CRISPR/Cas-based genome editing, showing the limitations of current transformation and regeneration protocols. After CRISPR/Cas reagents, such as a plasmid DNA vector or RNP for the genes of interest, are transformed into explant cells, these cells must then be regenerated into mutant transgenic plants. Each step of the tissue culture process reduces its efficiency owing to the regeneration of non-transformed plants and the regeneration of transformed plants that lack the desired edit. Antibiotics are typically added to the culture medium to increase the proportion of transformed cells by inhibiting the growth of non-transformed cells (only in the Agrobacterium T-DNA transfer method). The transformation efficiency, regeneration rate, and in vivo activity of CRISPR/Cas reagents all impact the total genome editing efficiency during this process. However, genome editing efficiency in most tree genome editing practices (Table 1) has not been accurately measured. Efficiency is typically calculated as A/(A+B+C), where A indicates the number of mutant transgenic plants, and B and C indicate the numbers of non-mutant transgenic plants obtained from transformed and non-transformed cells. This does not account for the number of explant cells that were transformed but not regenerated. Most tree genome editing studies have focused more on whether the CRISPR/Cas reagents function than on their efficiency. Conventional protocols for transformation and regeneration are laborious and time-consuming, and their low efficiencies are major obstacles to tree genome editing using the CRISPR/Cas system. Possible solutions to these problems are discussed in Section 4.

As shown in Table 1, targeted mutagenesis using the CRISPR/Cas system in trees still relies on the laborious and tedious processes of conventional protocols for the transformation of CRISPR/Cas reagents and the regeneration of transformants. Among conventional transformation protocols, *Agrobacterium* mediated transformation protocols are most efficient and have been most widely employed, but they have still been restricted to a few types of explants such as the juvenile leaf, petiole, cotyledon, or embryogenic cell masses within only a small percentage of tree species because many economically important tree species such as citrus trees are generally recalcitrant to *Agrobacterium* infection. Moreover, tissue culture systems have only been established for a limited number of tree species, with several species, such as *Theobroma cacao*, shown to be recalcitrant to this approach. Even for tree species with no tissue culture problems, transformation efficiency is far below 100%, resulting in significant regeneration of non-transgenic plantlets as well as transgenic plantlets that lack the desired edits (Fig. 1).

A selection process is required during the regeneration of transformants to ensure that non-transformed plants are not carried forward into subsequent steps. The primary selection method for *Agrobacterium*-mediated T-DNA transformation is antibiotic resistance-based selection, which works by inhibiting the growth of non-transformed cells. Culture media with antibiotics should only allow the growth of the transformants that possess antibiotic resistance genes present in the transformed T-DNA. However, the antibiotic resistance gene products of the transformed cells are secreted into the culture medium and degrade the antibiotics in their vicinity, allowing the growth of neighboring non-transformed cells, reducing selection efficiency. Reporter gene (*GUS* or *GFP*)-based selection cannot inhibit the growth of non-transformants and is therefore not ideal for the process. In transient transformation such as T-DNA-free RNP transfection, the selection to screen out mutant plantlets depends on PCR and sequencing of randomly chosen samples, resulting in much lower efficiency. Recently, a novel approach called transient CRISPR/Cas editing in plants (TCEP) has been developed to quantitatively determine the *in vivo* activity of CRISPR/Cas reagents during transient transformation^[57]^. Precise assessment of transient activity of CRISPR/Cas reagents by this approach will help to guide improvements in the process, enabling easier selection of genome-edited cells. Unfortunately, this process still depends on the extraction of DNA or RNA, which means that the tested cells cannot survive after the DNA or RNA extraction. Therefore, even if very high activity of Cas/sgRNAs is determined by the TCEP method, it is impossible to culture those cells and regenerate mutant plantlets from them. Overall, significant progress has been made in editing tree genomes, but efficiency is still low.

## Potential limitations of editing trees with the current CRISPR/Cas system

### Current systems for transient transformation are not sufficient for the efficient editing of tree species

Transformation systems deliver *in vitro* manufactured CRISPR/Cas reagents, such as plasmids or RNPs, into cells. After transformation, the CRISPR/Cas system is converted into an RNP complex of Cas nuclease and sgRNAs, which then edits the target loci. It should be noted that delivery of CRISPR/Cas reagents into the plant cell does not guarantee delivery into the nucleus owing to the compartmentalized nature of eukaryotic cell structures. All eukaryotic organelles, including the nucleus, are enclosed within membrane structures that can prevent movement of the complex. Because Cas nuclease editing requires entry into the nucleus, the presence of a nuclear envelope can significantly affect editing efficiency.

Delivery and integration of T-DNA into the genome of host plant cells by *Agrobacterium* has been the predominant transformation protocol, and its underlying mechanisms are well understood^[58]^. The transgenes (Cas nuclease gene and sgRNAs) in the T-DNA region that are integrated into the genome can be stably expressed in the nucleus. In transient transformation, non-integrated T-DNA strands duplicated by DNA polymerase θ are transiently expressed in the nucleus^[58]^, but the non-integrated T-DNA cannot be inherited by progeny cells. Cas nuclease mRNAs and sgRNAs are transcribed in the nucleus, and the mRNAs are then transferred to the cytoplasmic matrix to be expressed. Because the Cas9 nuclease protein must enter the nucleus to edit the genome, the NLS peptide is attached to it in order to allow its entry. This system therefore bypasses the barrier posed by the nuclear envelope during *Agrobacterium*-mediated transformation.

**Figure 2:**
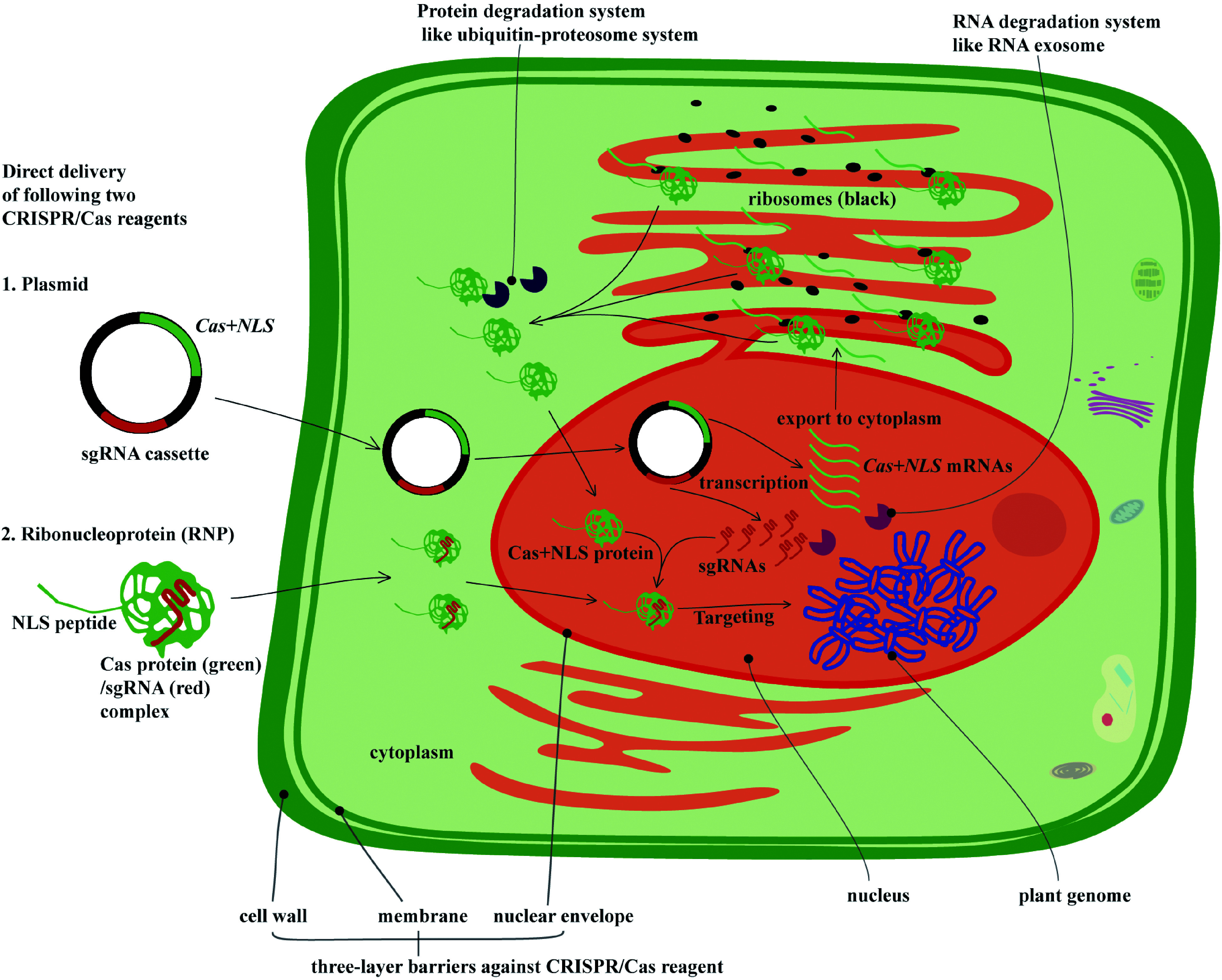
Direct delivery of CRISPR/Cas reagents (plasmid or RNP) and potential barriers affecting their delivery efficiency and intranuclear genome editing activities. The active form of CRISPR/Cas reagents is the RNP, which is generated from transcription and translation of the CRISPR/Cas and sgRNA sequences. Because transcription only takes place in the nucleus, these plasmids must therefore gain entry to this cellular compartment. In the nucleus, *Cas+NLS* and sgRNAs are transcribed into RNAs, and the *Cas + NLS* mRNA must be exported into the cytoplasm to be translated into the Cas + NLS protein, which then re-enters the nucleus to form the RNP complex with sgRNAs. Therefore, the plasmid delivery process involves a total of three passes through the nuclear envelope. Although this process has been studied extensively, it still remains unclear how the nuclear envelope regulates the import of plasmid DNA, RNA, or RNP complexes into the nucleus, and the low efficiency of direct delivery systems may be due to the negative regulatory role of the nuclear envelope during the nucleocytoplasmic transfer of CRISPR/Cas reagents into the nucleus. Furthermore, intracellular protein and RNA degradation systems, such as the Ubiquitin-Proteosome and RNA exosome, may be potential obstacles for the RNP complex. These “degradosomes” may render the activity of RNPs more transient, resulting in a much lower editing efficiency.

Other transient transformation protocols, such as biolistic bombardment or PEG-mediated delivery of CRISPR/Cas reagents through plasmids or RNPs, have also been employed in genome editing practices (Fig. 2). These protocols are regarded as more direct and straightforward than *Agrobacterium*-mediated transformation, but their efficiency in genome editing is lower (Table 1). This low efficiency may be due to the fact that such direct transformation systems can stall upon cell entry due to additional barriers, such as the nuclear envelope. Delivery of CRISPR/Cas reagents from the cytoplasm to the nucleus where the target genes are accessible is required, but the delivery mechanism of transient systems is still unclear, making their success unpredictable. Efficient transgene-free genome editing by direct delivery of plasmids or RNPs relies on effective nucleocytoplasmic transfer of the plasmid or RNP into the nucleus via pores in the nuclear envelope. Relatively low efficiency of genome editing in transient transformation using direct delivery of plasmids or RNPs implies that the nucleocytoplasmic transfer of CRISPR/Cas reagents into the nucleus may be a major limiting factor to efficient editing. Although the components and functions of nuclear envelope structures are well known^[59,60]^, the mechanism by which they affect the transfer of CRISPR/Cas reagents into the nucleus is still poorly understood. Mitosis during cell division may allow the import of CRISPR/Cas reagents into the nucleus^[61]^ because nuclear envelope breakdown and reorganization events occur during this time. This would present an opportunity for the import of CRISPR/Cas reagent into the nucleus, although it is unclear whether or not the CRISPR/Cas reagent would be compartmentalized inside the reorganized nucleus. The low efficiency of RNP transfer may also be due to other mechanisms, such as protein or RNA decay mediated by endogenous degradation systems^[62,63]^. Thus far, no reports are available on the stability of sgRNAs in the cytoplasm or the nucleus, hindering improvements in sgRNA stability. Only fusion of the NLS peptide into Cas nuclease to promote the delivery of CRISPR/Cas reagents into the nucleus (Table 1) and high concentrations of RNP complexes have been attempted to increase editing efficiency in tree genomes. Further research is required to understand the mechanisms of nucleocytoplasmic transfer and intracellular degradation of the plasmid DNA, small sgRNA, and Cas nuclease protein during transient transformation to stabilize CRISPR/Cas reagents and improve their delivery through the nuclear envelope.

### Process for regeneration of the mutated cells into mutant plantlets is time-consuming and laborious

**Figure 3:**
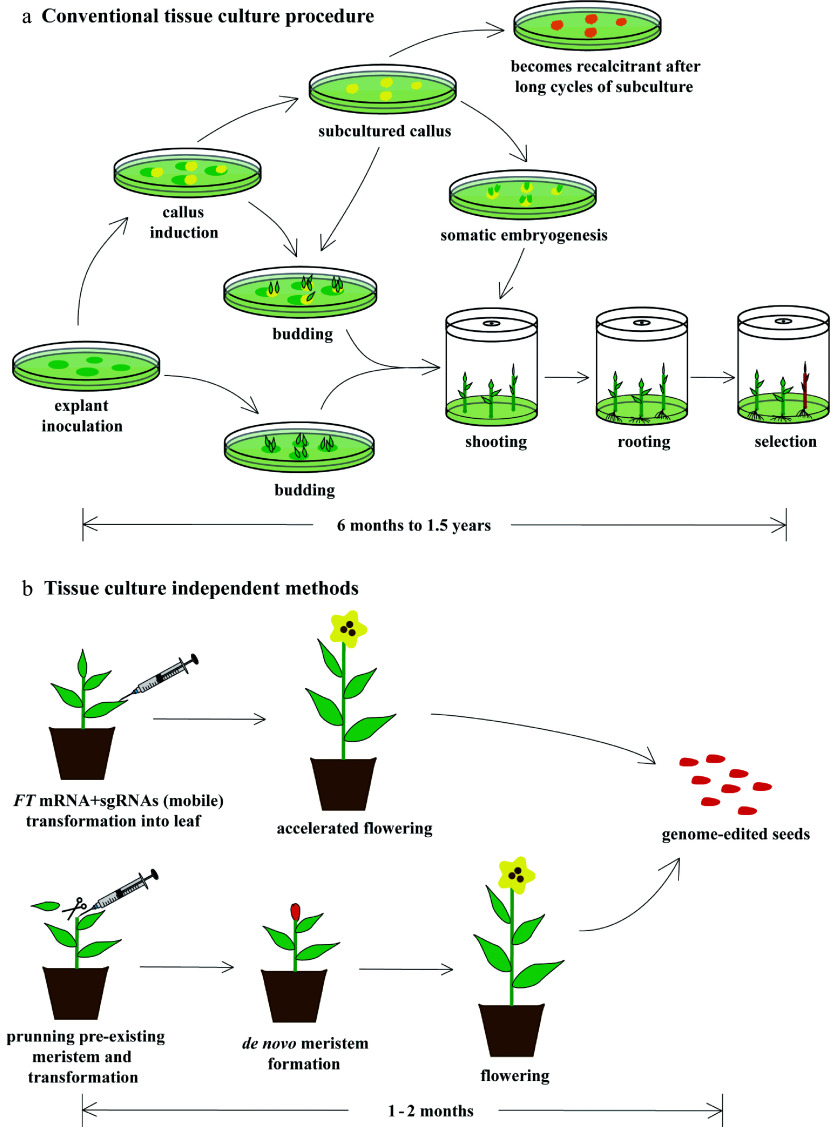
Faster and easier regeneration of genome-edited plants by tissue culture–independent protocols. (a) Conventional tissue culture is both tedious and laborious. This process normally takes anywhere from six to eighteen months and requires a sterile environment and a large amount of tissue culture medium, dishes, bottles, and chemical reagents. Its regeneration efficiency is relatively low, and recalcitrancy limits its utility. (b) Recently, novel technologies, such as mobilization of sgRNAs by *FT* mRNA fusion and de novo meristem induction, have been developed, enabling researchers to overcome some of the problems of conventional tissue culture. In the *FT* mRNA/sgRNAs protocol, *FT* mRNA encodes the mobile florigen essential for induction of flowering, which is fused to sgRNAs to facilitate their movement from the leaf to the shoot apical meristem. This causes genome editing of the floral meristem, which results in genome-edited seed production. In the de novo meristem induction protocol, genome editing and meristem induction are performed simultaneously to generate genome-edited seeds. These in planta transformation protocols require only one or two months to generate genome-edited plants. In addition, these protocols do not require laborious processes of sterilization and sterile tissue culture.

Tissue culture techniques, such as somatic embryogenesis, callus induction, and shoot and root organogenesis, have been used to clone mutant cells and regenerate mutant plantlets. However, these processes can take six months or longer to regenerate T0 or T1 transgenic mutant plants, during which extensive work must be carried out in order to continually select for positive transgenic plants using antibiotic resistance genes or reporter genes (Fig. 3a). Thus far, no work has been conducted to attempt to overcome these challenges in genome engineering of tree species (Table 1). In non-trees, such as herbs and crops, novel protocols have already been applied to establish time-saving strategies for genome editing. These protocols involve both tissue culture–dependent and tissue culture–independent strategies.

Among the tissue culture–independent methods, one protocol involves mobile sgRNAs that can move from the leaf to the shoot apical meristem (SAM) for tobacco genome editing^[64]^. In this process, sgRNA was fused with a *Flowering Locus T* (*FT*) mRNA encoding the mobile florigen essential for induction of flowering, and the result was termed "mobile sgRNA". This sgRNA was then transformed using tobacco rattle virus (TRV) into the leaf of a tobacco plant overexpressing the Cas9 nuclease. Transformed mobile sgRNA then moved from the leaf to the SAM and edited the target gene with high efficiency, while *FT* induced flowering and seed formation from the edited SAM, finally generating genome-edited seeds. This protocol relies on *in planta* transformation that exploits the natural developmental process from the SAM to flowers and then seeds. It also bypasses the time-consuming process of tissue culture but still achieves high efficiency. In addition, *FT* induces precocious flowering, thereby shortening the time for seed development and reducing the entire process to only one or two months. Another protocol utilizing *in planta* transformation through *Agrobacterium* has also achieved rapid and efficient genome editing of tobacco plants^[65]^. In this study, *de novo* reprogramming of somatic tissues into plant meristems (mainly SAMs) and genome editing were concurrently induced by *Agrobacterium-*mediated transformation of co-expression vectors containing both developmental regulators (DRs) and sgRNA cassettes into pruned sites of tobacco plants overexpressing Cas9 nuclease^[66]^. CRISPR/Cas then edited the genome of somatic cells, and DRs induced *de novo* reprogramming of genome-edited somatic cells into meristems, finally leading to fertile plants and genome-edited seed production. This *in planta* protocol enabled both rapid and efficient genome editing by omitting tissue culture. Although both of these novel approaches (Fig. 3b) are just the beginning of tissue culture–free genome editing and have some limitations^[64,65]^, they represent a significant step towards the simplification of CRISPR/Cas-mediated genome editing in plants. Because these protocols rely on the natural developmental process that proceeds from the SAM to flowers and then seeds, they can be applied to angiosperms such as poplars. Gymnosperms have different reproductive mechanisms^[67]^ and may therefore require some additional modification to enable the successful *in planta* transformation of CRISPR/Cas.

Other attempts to save time during crop improvement and research have shown that tissue culture–based regeneration can be boosted with the aid of speed breeding. The concept of speed breeding was first proposed by Watson et al. in 2018^[68]^ and is now regarded as a promising technique for accelerating crop breeding and improvement. In speed breeding, techniques for the regulation of multiple factors, including photoperiod, light intensity, temperature, moisture, high cultivation density, and plant hormones, have been harnessed to shorten the time to reproduction^[68,69]^. Harvesting and germinating immature seeds are also crucial for reducing the generation time^[68]^. Speed breeding greatly reduces generation time, thereby allowing the production of 3 to 9 generations per year in various plant species including *Arabidopsis*, barley, chickpea, rice, soybean, and wheat^[70−74]^. These experiences from speed breeding practices can be exploited for tissue culture practices to save time in tree regeneration. In addition to speed breeding, genetic manipulations can also boost regeneration speed. Recently, GROWTH-REGULATING FACTOR (GRF) and its cofactor GRF-INTERACTING FACTOR (GIF), as well as GRF-GIF chimera, have been shown to improve regeneration efficiency in plant transformation and genome editing practices^[75,76]^. The GRF-GIF chimera has several advantages. First, its mode of action is different from the aforementioned *de novo* SAM induction by DRs such as BBM and WUS transcription factors, and it can therefore be used to avoid the side-effects of those systems^[66,77]^. In addition, the GRF-GIF chimera can act as a reporter gene, eliminating the need for an antibiotic resistance marker. For example, its transformation into callus generates new green embryos, enabling easy identification of transformed tissues^[76]^. Finally, GRF-GIF chimera solves issues related to callus regeneration and have been shown to enable the regeneration of recalcitrant callus in wheat^[76]^. Results suggest that the GRF-GIF chimera enables the successful transformation of CRISPR/Cas reagents and the subsequent regeneration of even recalcitrant plants, making it especially useful for tree genome editing.

As described above, several innovative protocols have emerged to achieve efficient regeneration of genome-edited seeds or plantlets via tissue culture–independent or tissue culture–dependent pathways. Their application has thus far been restricted to *Arabidopsis*, tobacco, and a few crop species, and some additional improvements are needed before their widespread deployment. Despite these limitations, they represent a significant step forward and could potentially be used to overcome the unique challenges of editing tree species.

### More data on the outcomes of tree genome editing are needed to facilitate its improvement

Current studies on tree genome editing focus primarily on the induction of target-specific DSBs. After the transformation of Cas9/sgRNA constructs into cells, the sgRNAs guide the nuclease toward the target locus of the genome to induce the DSBs. DNA repair pathways then result in several different mutagenesis outcomes. Most tree genome editing studies have only assessed the induction of DSBs and subsequent mutagenesis, and more data are needed to understand the mechanism by which Cas9/sgRNA-induced DSBs generate biallelic, homozygous, heterozygous, or chimeric mutations in tree genome editing.

Although DSBs induced at the target loci initiate the editing process, they are not directly responsible for differences in the resulting mutants. The DSBs are recognized as genotoxic lesions, and consequently, intrinsic DNA repair pathways, such as homologous recombination (HR), classical nonhomologous end joining (cNHEJ), microhomology-mediated end joining (MMEJ), and single-strand annealing (SSA), are activated to repair the DSBs. Among these pathways, only the template-dependent HR is error-free, and others, including template-independent cNHEJ, MMEJ, and SSA, are error-prone. Therefore, the accuracy and efficiency of targeted mutagenesis are greatly influenced by which DNA repair pathway is activated. However, the underlying causes of repair mechanism selection are not clear, making it impossible to predict which one will be activated after Cas9 cleavage^[78]^.

Emerging evidence indicates that Cas9 nuclease–induced DSB repair results in the human genome are not random^[79−81]^. Based on the nonrandom nature of DSB repair, machine learning algorithms and abundant experimental data of repair outcomes have been combined to predict outcomes^[80−82]^. Three machine learning (ML) models, inDelphi, FORECasT, and SPROUT, are typically employed for predicting Cas9-induced DSB repair results. ML models have continued to develop, and a new method called CROTON is highly automated and simplified by an end-to-end framework with better results than earlier algorithms^[83]^. These ML models enable precise prediction of mutagenesis outcomes without the need to conduct wet-lab experiments, thereby saving large amounts of time, effort, and reagents. However, none of these models has yet been trained on tree cells, and model training requires abundant data on DSB-induced DNA repair outcomes obtained from wet-lab experiments of tree genome editing using diverse sgRNAs.

## Potential applications of newly emerging CRISPR/Cas toolkits to trees

To date, various Cas nucleases such as Cas12a (Cpf1), Cas13, and Cas14 have been developed and applied to genome editing in mammals and plants^[6−8]^. In trees, genome editing practices have used mostly Cas9 (Table 1). Cas12a, Cas13, and Cas14 have several advantages over standard Cas9, such as additional RNA cleavage activity and diverse PAM profiles; they enable RNA editing as well as single stranded RNA/DNA targeting, expanding genome editing toolkits and their applicability. Also, they are smaller than Cas9, promoting their entry into the nucleus and broadening the range of selectable vectors. Wild-type Cas nucleases have also been modified into nuclease-deactivated Cas proteins (dCas nucleases such as dCas9 or dCas12), which are then tethered to various effector proteins and harnessed to achieve a broad range of applications, such as CRISPR interference (CRISPRi), CRISPR activation (CRISPRa), and epigenome editing^[84−88]^. Recent progress in their mechanisms and applications in human and plants have been the subject of several reviews^[89−92]^. Thus far, only a few studies have reported the use of Cas12a^[23,53]^ and CRISPRi^[19]^ in trees, and further application of the various Cas nucleases and dCas/effector complexes will undoubtedly expand the versatility and efficiency of CRISPR/Cas genome editing toolkits in trees.

In addition, the dCas nucleases have been tethered to effector proteins like base editors and prime editors, thereby enabling base editing or prime editing while overcoming the limitations of classical CRISPR/Cas systems^[3]^. Classical CRISPR/Cas systems have successfully achieved precise targeting, and the resulting outcomes mainly reflect indel mutagenesis (Table 1) by DSBs and subsequent error-prone DNA repair pathways such as nonhomologous end joining (NHEJ). Mutation outcomes from NHEJ repair pathways are now subject to prediction, as mentioned in Section 4.3, but the outcomes typically include a large number of undesired changes, thus reducing the precision of genome editing. Error-free HR pathways and donor DNA-dependent homology directed repair (HDR) offer the potential for precise genome editing. However, in the DSB-induced repair process, the HDR pathway competes with error-prone DNA repair pathways, and the efficiency of precise genome editing is therefore very low, limiting the application of this approach^[93,94]^. Classical CRISPR/Cas genome editing tools using DSBs have significantly advanced, but they reveal their limitations when dealing with SNPs, which are not only important pathogenic point mutations in human but also agronomically important genetic variations. Novel CRISPR/Cas toolkits such as base editing and prime editing have recently emerged as alternative genome editing tools. They have enabled efficient, versatile, and precise editing by installing or reverting transition/transversion point mutations and even directly copying desired sequences into targets in mammals, plants and bacteria^[95−97]^. They use the nuclease-deactivated dCas9 protein fused with base editors (adenosine deaminase and/or cytidine deaminase fused with uracil DNA glycosylase) or prime editors (reverse transcriptase fused with a prime editing guide RNA; pegRNA), enabling efficient and precise genome editing without the need for DSBs or donor DNA templates^[95]^. Base editing and prime editing were reported in mammals such as human and mice in 2016^[98]^ and 2019^[96]^, respectively, and then applied to crop plants such as rice and wheat, opening up new possibilities for plant genome editing^[99,100]^. Although they have not yet been applied to trees, they have great potential for rapidly accelerating tree breeding and trait improvement. For example, trees typically have highly abundant SNPs—as many as one SNP per 100 bp in their genomes. SNPs in genes lead to changes in gene activities, thereby causing phenotypic changes associated with plant growth and development and responses to abiotic and biotic stress. Because of the high abundance of SNPs in tree genomes of different species, identifying functional SNPs and determining their roles through phenotypic validation are urgent tasks for tree breeding and trait improvement and require large numbers of tree SNP models. Novel CRISPR/Cas toolkits such as base editing and prime editing can create tree models that carry desired SNPs precisely and efficiently.

## Conclusions

The CRISPR/Cas system has been used for targeted genome editing of trees since 2014. Despite natural barriers, including large genome sizes, high polyploidy and heterozygosity, and abundant SNPs, rapidly developing tree genome data and sgRNA design tools have enabled successful targeted genome editing in several tree species. Over the last seven years, the CRISPR/Cas system has been successfully applied to many tree species, including apple, bamboo, Cannabaceae, cassava, citrus, cacao tree, coffee tree, grapevine, kiwifruit, pear, pomegranate, poplar, ratanjoyt, and rubber tree. CRISPR/Cas-based mutagenesis at desired target loci has been demonstrated in these species, contributing to the further development of genome editing in trees and enabling the identification of genes associated with tree growth, secondary metabolism, and resistance to biotic and abiotic stress. However, genome editing still has several limitations, and most practices have relied on high-efficiency *Agrobacterium*-mediated stable transformation, which is not favorable in the current regulatory environment. Transient transformation protocols, such as the delivery of RNP complexes, can achieve transgene-free (non-GMO) genome editing and are preferred. However, the efficiency of such systems is currently very low, limiting their widespread application^[12,55]^.

Low transformation efficiency is the main factor limiting the application of transient CRISPR/Cas systems for genome editing in trees. Lack of knowledge about the intracellular stability and nucleocytoplasmic delivery of CRISPR/Cas reagents (plasmid DNA or RNP) hampers efforts made to improve this system. In addition, low regeneration efficiency results in a significant waste of time, effort, and reagents, creating further challenges for CRISPR/Cas-based genome editing in trees. Several innovations have emerged to promote efficient regeneration of genome-edited seeds or plantlets with or without the need for tissue culture. Although these approaches have not yet been applied to tree species, they represent new avenues for improving the efficiency and simplicity of tree genome editing. In addition, the DSBs created by Cas9 cleavage are known to result in the activation of different repair pathways that generate different outcomes. Machine learning models are now being used for effective prediction of mutagenesis outcomes, but they still require the input of large amounts of empirical data, which are currently unavailable for trees.

CRISPR/Cas genome editing practices in trees have thus far relied mainly on the classical wild-type Cas9 nuclease. Other wild-type Cas nucleases such as Cas12a (Cpf1), Cas13, and Cas14 and dCas nucleases tethered to various effectors, including transcriptional regulators and epigenetic modifiers, are newly emerging CRISPR/Cas toolkits that can be used for a broad range of applications beyond basic genome editing, including CRISPRi, CRISPRa, and epigenome editing. dCas nucleases tethered to base editors or prime editors can also be harnessed to improve the precision of genome editing practices, an approach that shows great potential for the generation of tree SNP models.

CRISRP/Cas system-based tree genome editing is still evolving and requires innovations in conventional transformation and regeneration protocols, as well as machine learning model-based simulation of mutagenesis to achieve more efficient and rapid outcomes. *In planta* transformation and tissue culture–free or modified tissue culture protocols, which have been developed recently, show great potential to improve the efficiency of CRISPR/Cas toolkits. Together with these novel strategies for transformation and regeneration, newly emerging CRISPR/Cas toolkits show great versatility, and their application to trees will expedite tree breeding and trait improvement.

## SUPPLEMENTARY DATA

Supplementary data to this article can be found online.
