# Supplementary material for: Progress and challenges in applying CRISPR/Cas techniques to the genome editing of trees
Source: For Res (Fayettev). 2022 May 11;2:6. doi: 10.48130/FR-2022-0006 (PMC11524270; doi:10.48130/FR-2022-0006)
Supplement: Supplementary file 1 — Supplementary data to this article can be found online. [file FR-2022-0006-Suppl-TableS1.docx]

**Supplemental Information**

**Supplemental Table S1. Current availability of tree genome data in sgRNA design tools**

| **Tree species** | **CRISPR RGEN**  **(Cas-OFFinder&**  **Cas-Designer)** | **CRISPOR** | **CRISPR-P 2.0** | **CHOPCHOP** | **CRISPRdirect** | **CCTOP** | **E-CRISP** |
| --- | --- | --- | --- | --- | --- | --- | --- |
| *Actinidia chinensis* | A | A | A |  | A |  |  |
| *A. eriantha* |  | A |  |  |  |  |  |
| *Carica papaya* |  | A |  |  | A | A |  |
| *Citrus clementina* | A |  |  |  | A |  |  |
| *C. sinensis* | A |  | A |  | A | A |  |
| *Coffea canephora* | A |  | A | A | A |  |  |
| *Diospyros kaki* |  |  | A |  |  |  |  |
| *Eucalyptus grandis* | A |  |  | A | A | A |  |
| hybrid poplar clone 717 | A |  |  |  |  |  |  |
| *Juglans microcarpa* | A |  |  |  |  |  |  |
| *Juglans regia* | A |  |  |  |  |  |  |
| *Malus domestica* | A | A | A |  | A | A |  |
| *Manihot esculenta* | A |  | A |  | A | A |  |
| *Musa acuminata* | A | A | A |  | A |  |  |
| *Populus alba* (sPta717 v2) |  |  |  |  |  | A |  |
| *Populus alba x Populus tremula* var. glandulosa | A |  |  |  |  |  |  |
| *Populus deltoides* | A | A |  | A |  |  |  |
| *Populus tremula* | A |  |  |  |  | A |  |
| *Populus tremula x alba* | A |  |  |  |  |  |  |
| *Populus tremula x tremuloides* | A |  |  |  |  |  |  |
| *Populus tremuloides* | A |  |  |  |  |  |  |
| *Populus trichocarpa* | A | A | A | A |  | A | A |
| *Prunus avium* | A |  | A |  |  |  |  |
| *Prunus persica* | A | A |  |  | A |  |  |
| *Pyrus x bretschneideri* | A |  |  |  |  |  |  |
| *Pyrus communis* |  | A |  |  |  |  |  |
| *Ricinus communis* |  |  |  |  | A |  |  |
| *Theobroma cacao* | A | A |  |  | A |  |  |
| *Vitis vinifera* | A |  | A |  | A |  | A |
| Uploading genome data for new species by sending request | A | A |  | A | A | A |  |

‘A’ indicates available or attainable, while blank indicates non-available or not attainable.

Websites for the tools are as follows.

1. Cas-OFFinder: <http://www.rgenome.net/cas-offinder/>

Cas-Designer: <http://www.rgenome.net/cas-designer/>

1. CRISPOR: <http://crispor.tefor.net/>
2. CRISPR-P 2.0: <http://crispr.hzau.edu.cn/CRISPR2/>
3. CHOPCHOP: <https://chopchop.cbu.uib.no/>
4. CRISPRdirect: <http://crispr.dbcls.jp/>
5. CCTOP: <https://cctop.cos.uni-heidelberg.de/species.html>
6. E-CRISP: <http://www.e-crisp.org/E-CRISP/>
